# Consistent Prebiotic Effects of Carrot RG-I on the Gut Microbiota of Four Human Adult Donors in the SHIME^®^ Model despite Baseline Individual Variability

**DOI:** 10.3390/microorganisms9102142

**Published:** 2021-10-13

**Authors:** Pieter Van den Abbeele, Cindy Duysburgh, Ilse Cleenwerck, Ruud Albers, Massimo Marzorati, Annick Mercenier

**Affiliations:** 1 ProDigest, 9052 Ghent, Belgium; cindy.duysburgh@prodigest.eu (C.D.); massimo.marzorati@prodigest.eu (M.M.); 2 Cryptobiotix, 9052 Ghent, Belgium; pieter.vandenabbeele@cryptobiotix.eu (P.V.d.A.); 3 BCCM/LMG Bacteria Collection, Laboratory of Microbiology (L.M.), Ghent University, 9000 Ghent, Belgium; Ilse.Cleenwerck@UGent.be; 4 Nutrileads, Nutrileads BV, 6708 WH Wageningen, The Netherlands; ruud.albers@nutrileads.com; 5 Center of Microbial Ecology and Technology (CMET), Ghent University, 9000 Ghent, Belgium

**Keywords:** pectin, rhamnogalacturonan I, *Bifidobacteriaceae*, *Bacteroides dorei*, acetate, propionate, in vitro

## Abstract

The human gut microbiome is currently recognized to play a vital role in human biology and development, with diet as a major modulator. Therefore, novel indigestible polysaccharides that confer a health benefit upon their fermentation by the microbiome are under investigation. Based on the recently demonstrated prebiotic potential of a carrot-derived pectin extract enriched for rhamnogalacturonan I (cRG-I), the current study aimed to assess the impact of cRG-I upon repeated administration using the M-SHIME technology (3 weeks at 3g cRG-I/d). Consistent effects across four simulated adult donors included enhanced levels of acetate (+21.1 mM), propionate (+17.6 mM), and to a lesser extent butyrate (+4.1 mM), coinciding with a marked increase of OTUs related to *Bacteroides dorei* and *Prevotella* species with versatile enzymatic potential likely allowing them to serve as primary degraders of cRG-I. These *Bacteroidetes* members are able to produce succinate, explaining the consistent increase of an OTU related to the succinate-converting *Phascolarctobacterium faecium* (+0.47 log_10_(cells/mL)). While the *Bifidobacteriaceae* family remained unaffected, a specific OTU related to *Bifidobacterium longum* increased significantly upon cRG-I treatment (+1.32 log_10_(cells/mL)). Additional monoculture experiments suggested that *Bifidobacterium* species are unable to ferment cRG-I structures as such and that *B. longum* probably feeds on arabinan and galactan side chains of cRG-I, released by aforementioned *Bacteroidetes* members. Overall, this study confirms the prebiotic potential of cRG-I and additionally highlights the marked consistency of the microbial changes observed across simulated subjects, suggesting the involvement of a specialized consortium in cRG-I fermentation by the human gut microbiome.

## 1. Introduction

Over the past decades, it became clear that the human gut microbiome constitutes an overlooked system that makes a significant contribution to human biology and development [1]. Observational studies applying metabolomics and metagenomics have largely broadened our understanding on how the human gut microbiota is associated with, among other things, obesity-related diseases [2], liver diseases [3], inflammatory bowel disease (IBD) [4], and colorectal cancer [5]. A key function of the gut microbiome consists in the fermentation of carbohydrates in the colon, resulting among other things in the production of short-chain fatty acids (SCFA) [6], mainly including acetate, propionate, and butyrate, which have each been related to particular health benefits as reviewed elsewhere [7,8,9,10]. Among the hundreds of gut microbes, *Bacteroides* species display diverse and versatile glycan metabolising capabilities, allowing them to ferment extremely complex glycans [11,12,13,14], with other members such as *Bifidobacterium* species growing on the polysaccharide degradation products and metabolites [15]. In contrast to the beneficial effect of fiber fermentation, fermentation of amino acids results in the formation of potentially detrimental compounds (e.g., phenols, cresol, and hydrogen sulfide), contributing to IBD or colon cancer [16]. Due to the purported role of dietary fiber and plant-based foods to inhibit such detrimental effects, there is a good rationale for developing functional foods that improve gut health via their impact on the gut microbiome [1]. 

Besides probiotics and polyphenols, prebiotic carbohydrates that are defined as substrates that are selectively utilized by beneficial host microorganisms [17] are widely studied for their health-promoting properties. While fructans such as fructo-oligosaccharides [18] and inulin [19] have well-established prebiotic effects, novel classes of prebiotics include, amongst others, human milk oligosaccharides [20], arabinoxylans [21], and a specific class of pectin-derived polysaccharides such as rhamnogalacturonan-1 (RG-I) [22,23,24]. The backbone of RG-I consists of repeated units of the disaccharide [-4)-α-D-galacturonic acid-(1,2)-α-L-rhamnose-(1] with sidechains comprising mainly D-galactose (~galactans), L-arabinose (~arabinans), and D-xylose (~xylans) branching off the rhamnose residues [23]. These sidechains, varying in composition and length, offer RG-I a structural complexity that requires the concerted action of different enzymes provided by a microbial consortium to be fermented [25]. The development and demonstration of functionality of a particular source of RG-I relevant for the current paper, i.e., carrot-derived RG-I (cRG-I), was recently reviewed by McKay et al. [26]. A clear immunostimulatory activity could be attributed to cRG-I both in vitro and in vivo, while in vitro studies demonstrated its capacity to modulate the human gut microbiota [27].

The lack of access to the in vivo site of activity combined with SCFA being rapidly absorbed [28] makes it very difficult to draw meaningful conclusions on SCFA production in vivo unless complex techniques such as a stable-isotope dilution method are applied [29]. The use of in vitro models has been recognized as an established solution to overcome this issue [30]. These models range from fecal batch incubation strategies [27,31] to pH-controlled reactor systems [32,33,34,35], each having their distinct benefits and potential drawbacks. An important aspect to take into account in gut microbiome research is the currently well-established considerable inter-individual differences among donors [36], which can greatly impact the results of dietary interventions [37] or in vitro outcomes of fiber fermentation [31]. In vitro studies should thus ideally include several test donors.

This study aimed to investigate the impact of repeated administration of cRG-I on the luminal and mucosal gut microbiota, while addressing potential interindividual differences among subjects. This complements the data of an earlier study where cRG-I treatment was evaluated over 2 days in batch fermentations [27]. cRG-I has earlier been shown to display immune modulation properties in vitro and to stimulate the innate immune response in healthy human subjects [26]. The data described in this paper thus support that cRG-I exerts a dual mode of action by impacting both the host immune system and gut microbiota. 

## 2. Materials and Methods

### 2.1. Chemicals

All chemicals were obtained from Sigma (Bornem, Belgium) unless otherwise stated. cRG-I (same substrate as tested in [27]) was supplied by Nutrileads (Wageningen, The Netherlands). The extract is highly enriched (80%) in the RG-I domain of pectin. Details on extraction method and extract characterization have been published in [26]. The monosaccharide composition of cRG-I is (% mol/mol): rhamnose, 14.3; arabinose, 34.8; galactose, 19.6; fucose, 0.8; glucose, 4.3; mannose, 0.9; xylose, 0.7; galacturonic acid, 25. 

### 2.2. Mucosal Simulator of the Human Intestinal Microbial Ecosystem (M-SHIME^®^)

**Figure 1:**
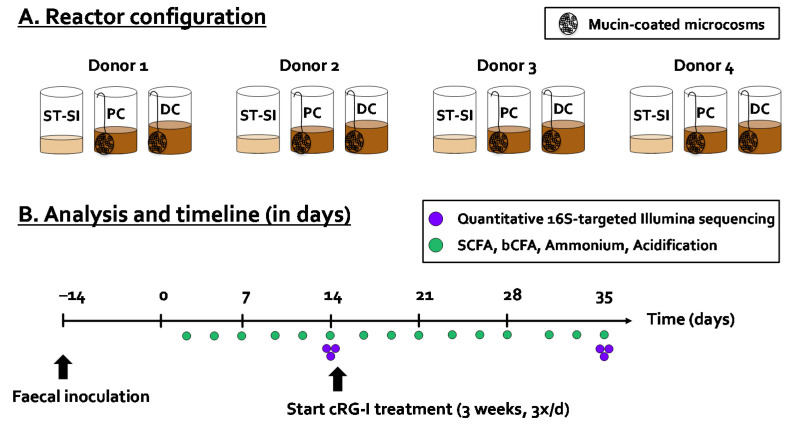
(**A**) Reactor configuration and (**B**) timeline together with performed analysis of quad-M-SHIME experiment during which the potential prebiotic effect of cRGI was investigated for four human adult donors (1, 2, 3, and 4). bCFA = branched-chain fatty acids, cRG-I = carrot-derived Rhamnogalacturonan I, DC = distal colon, PC = proximal colon, SCFA = short-chain fatty acids, quad-M-SHIME = Mucosal Simulator of the Human Intestinal Microbial Ecosystem with four parallel units. ST-SI = stomach–small Intestine.

The computer-controlled, automated SHIME^®^ technology (ProDigest, University of Ghent, Ghent, Belgium) was used to conduct the current study. In terms of reactor design, the classical M-SHIME^®^ setup was modified to allow parallel comparison of fermentation by four fecal samples, leading to a quad-M-SHIME setting, i.e., four parallel units, each comprising three sequential reactors (Figure 1A). The first reactor was a combined stomach/small intestinal reactor (ST/SI), while the other reactors simulated the proximal colon (PC) and distal colon (DC), respectively. In contrast to the ST/SI reactor that was operated according to a fill-and-draw principle, the PC and DC reactors had a fixed volume of 500 and 800 mL, respectively. The pH of the PC and DC was controlled within fixed intervals (at 5.7–5.9 and 6.6–6.9, respectively) via the addition of 0.5M NaOH and 0.5M HCl. Besides a luminal phase, each colonic reactor also contained 60 mucin-coated microcosms (AnoxKaldnes K1carrier, AnoxKaldnes AB, Lund, Sweden) prepared according to Van den Abbeele et al. [38] to allow simulation of the mucosal microbiota. 50% of the mucin beads were renewed three times per week (i.e., on Monday, Wednesday, and Friday) by opening the reactors as described recently [39]. All reactors were airtight, continuously agitated (300 rpm), and temperature controlled (37 °C). Anaerobiosis was maintained by flushing the headspace of all reactors once per day with nitrogen gas (Parker Balston Nitrogen Generator UHPN2-1100, CompAir Geveke, Vilvoorde, Belgium).

The experiment consisted of a stabilization (Day −14 to 0), control (Day 0 to 14), and cRG-I treatment period (Day 14 to 35) (Figure 1B). At day −14, the PC and DC reactors of each of the four units were filled with 500/800mL SHIME nutritional medium (ProDigest, Belgium) and inoculated with a fecal slurry (5% *v*/*v*) of one of the four donors under investigation (Donor 1 (f, 29), 2 (m, 27), 3 (f, 32), or 4 (f, 33)), prepared according to Moens et al. [40]. All donors were healthy with a normal BMI and did not use antibiotics during the last 6 months prior to this study. Each arm received three feeding cycles per day of 140 mL of SHIME nutritional medium. During the simulation of the small intestine, 60 mL of bile/pancreatic fluid (oxgall (6 g/L), sodium bicarbonate (12.5 g/L), and porcine pancreatin (0.9 g/L)) was added to the reactor. The content of the reactor was then pumped to PC and to the DC. Excess liquid was removed from the DC reactor to the waste vessel. While the SHIME^®^ was operated according to its standard parameters during the stabilization and control period (Day −14 to 0 and Day 0 to 14), cRG-I was added at 7.14 g/L in the nutritional medium between Days 14 to 35. Upon repeated administration (three times per day) and upon dilution with bile/pancreatic juice during each feeding cycle (140/200 ratio), this would thus result in a theoretical maximal concentration of 5 g/L in the PC and DC region.

To ensure proper operation of the SHIME model and avoid potential issues with stability over time, quality control was performed according to in-house procedures. Briefly, this involved a weekly verification of the accuracy of pumped volumes via a mock feeding cycle (allowing to verify the accuracy of pumped nutritional medium and bile/pancreatic fluid) together with a weekly check of the pH value inside the reactors via an external pH probe (Senseline pH meter F410 (ProSense, Oosterhout, The Netherlands).

### 2.3. Microbial Activity Analysis

The SHIME software (ProDigest, Ghent University, Ghent, Belgium) allowed for the online monitoring of base (0.5M NaOH) and acid (0.5 M HCl) consumption that was required to control the pH within the desired interval (5.7–5.9 for PC and 6.6–6.9 for DC). Therefore, a first indication on microbial activity was obtained via calculating the ‘base–acid consumption’ (mL/day). Short-chain fatty acid (SCFA; acetate, propionate, and butyrate) and branched-chain fatty acids levels (bCFA: sum of isobutyrate, isovalerate, and isocaproate) were measured using the GC-FID method described by De Weirdt et al. [41]. This method involves the extraction of SCFA from samples with diethyl ether after the addition of 2-methyl hexanoic acid as an internal standard. Finally, ammonium was quantified via steam distillation, followed by titrimetric determination with HCl [42].

### 2.4. Microbial Composition Analysis

DNA was extracted from pelleted bacterial cells derived from a 1 mL sample as described by Boon et al. [43] with modifications implemented by Duysburgh et al. [44]. Samples were collected on the final day of the control and treatment periods. Microbial community profiling via 16S-rDNA targeted Illumina sequencing (LGC Genomics GmbH, Berlin, Germany) was performed as described recently [27]. As described in [45,46], the sequencing analysis was adapted from the MiSeq protocol for read assembly and cleanup using the mothur software (v. 1.39.5) according to following procedures. First, reads were assembled into contigs, followed by alignment-based quality filtering via alignment to the mothur-reconstructed SILVA SEED alignment (v. 123). Upon removing chimeras, taxonomy was assigned via a naïve Bayesian classifier [47] and RDP release 14 [48]. Finally, contigs were clustered into OTUs at 97% sequence similarity. Across samples, the total number of raw reads was on average 75,210 (minimum = 22,312; maximum = 144,386), while the final number of combined reads was on average 35,116 (minimum 10484; maximum 66,947). Rarefaction curves were made with the software Past 4.03 [49] to confirm that the sequencing depth allowed to grasp the microbial diversity of samples of the four investigated regions (lumen and mucus of simulated proximal and distal colon). For each OTU, representative sequences were selected as the most abundant sequence within that OTU. The obtained results were presented as proportional values (accounting for the total amount of sequences within each sample) at different taxonomic entities (phylum, family, and OTU level). The proportional phylogenetic information was combined with an accurate quantification of the total amount of cells via flow cytometry to obtain a quantification of each of the aforementioned three taxonomic levels. Flow cytometry was performed as described by Van den Abbeele et al. [27]. Briefly, upon dilution in Dulbecco’s Phosphate-buffered Saline (DPBS) (Sigma–Aldrich, Bornem, Belgium) and staining with 0.01 mM SYTO24 (Life Technologies Europe, Merelbeke, Belgium), samples were analyzed on a BD Facsverse (BDBiosciences, Erembodegem, Belgium) and analyzed using FlowJo, version 10.5.2. The strength of combining sequencing data with cell counts is that samples with different cell densities can be compared more optimally [50]. It should be noted that the data obtained after multiplying sequencing data (%) with cell numbers derived from flow cytometry (cells/mL) result in an amount of a given taxonomic group (cells/mL). This should be considered as an estimated amount given that the output of the sequencing analysis in reads is also only providing an estimated abundance since one microbial cell can have multiple copies of the 16S rRNA gene and the number of copies can also differ between microbial species.

### 2.5. Bifidobacteria Growth Experiments on cRG-I

An additional experiment was performed to assess the potential growth of 20 *Bifidobacterium* strains on cRG-I (Table S2). For this purpose, the various strains were grown on LMG medium 144 agar (composition (g/L): special peptone (23), soluble starch (1), NaCl (5), cysteine hydrochloride (0.3), glucose (5), agar (15)) containing glucose (positive control), without glucose (negative control), or with cRG-I (0.5% *w/v*) instead of glucose (*n* = 2). Per plate, 2 × 50 µL of cell suspension was inoculated. Inocula were prepared by suspending cells from 48 h old cultures from the second or third generation on LMG medium 144 in saline (0.85% *w*/*v*). Results were read after 72 h of incubation at 37 °C under anaerobic conditions. Growth on cRG-I was scored after visual comparison to the positive and negative controls by one and the same researcher.

### 2.6. Statistics

For exploratory data analysis, principal component analysis (PCA) was performed for both metabolic (acidification, acetate, propionate, butyrate, bCFA, and ammonium) and compositional data (10 most abundant families as detected via 16S rDNA-targeted Illumina sequencing across all samples, i.e., *Bifidobacteriaceae*, *Bacteroidaceae*, *Prevotellaceae*, *Acidaminococcaceae*, *Lachnospiraceae*, *Ruminococcaceae*, *Veillonellaceae*, *Desulfovibrionaceae*, *Synergistaceae,* and *Akkermansiaceae*) via the online tool http://biit.cs.ut.ee/clustvis/ (accession date: 06/05/2021) [51]. The averages of each of the four donors were calculated for each readout, after which paired two-sided t-tests were performed to identify significant effects valid across donors. As there were, respectively, two and four comparisons for metabolic (=within PC and DC) and compositional endpoints (=within PC and DC, both for lumen and mucus), multiplicity was corrected using the Benjamini–Hochberg false discovery rate (FDR, with FDR = 0.10 for metabolic markers and FDR = 0.20 for 16S-targeted Illumina data) [52]. All calculations were carried out in Excel, while figures were prepared in the GraphPad Prism v9.1.1 software.

## 3. Results

### 3.1. cRG-I Consistently Stimulated Microbial Activity in the Simulated Proximal and Distal Colon of Four Human Adults Tested in the Quad-M-SHIME

**Figure 2:**
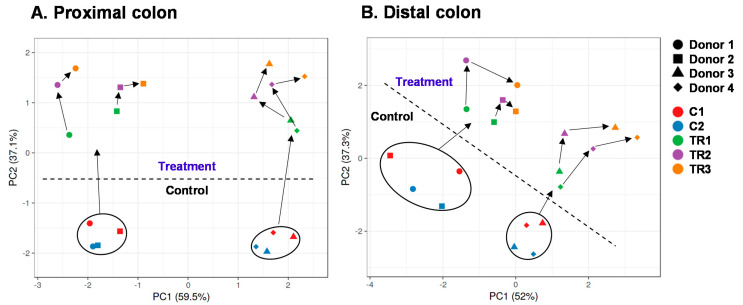
Principal component analysis (PCA) based on microbial metabolic activity in the proximal (**A**) and distal (**B**) colon during the control and treatment with cRG-I, as tested for four human adult donors 1, 2, 3, and 4 in the quad-M-SHIME. The PCA is based on the average values for each donor and for each experimental week (5 weeks: 2 control weeks (C1/C2) and 3 treatment weeks (TR1/TR2/TR3)). The following data were included: acidification, acetate, propionate, butyrate, ammonium, and bCFA. Arrows indicate the evolution of samples along the duration of the experiment. bCFA = branched-chain fatty acids, cRG-I = carrot-derived Rhamnogalacturonan I.

**Figure 3:**
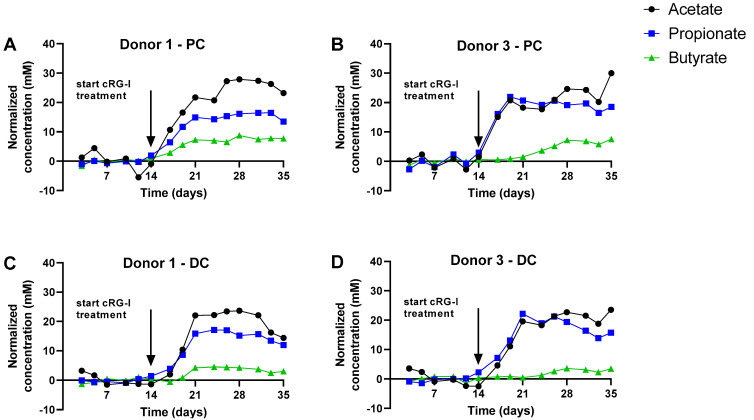
Effect of fermentation of cRG-I on SCFA production in the simulated proximal and distal colon during both the control (Days 0–14) and treatment period (Days 14–35) of the quad-M-SHIME for Donors 1 (panels **A** and **C**) and 3 (panels **B** and **D**). Data are expressed as normalized values versus the average of the control period. Donor A and C results are representative of those obtained for Donors B and D, respectively (see Supplementary Figure S1). Arrows indicate the start of the cRG-I treatment. cRG-I = carrot-derived Rhamnogalacturonan I, DC = distal colon, PC = proximal colon. The absolute values on which the normalized concentrations are based are shown in Figure S2 panels A/C/E/G.

To gain insight into overall changes of microbial activity upon cRG-I treatment, the data were presented in two ways: (i) PCAs (Figure 2) and (ii) longitudinal SCFA levels as normalized versus the control period (Figure 3 and Figure S1). Additionally, non-normalized data are presented as supplementary information (Figure S2). 

First, the PCAs based on the metabolic data explained a great part of the overall variation, i.e., 95.6% and 89.3% for the PC and DC, respectively. This observation stressed that both PCAs provide optimal insight in the factors underlying variation. A first factor included the interindividual differences among the four donors tested. Already at baseline, the samples of donors 1/2 and donors 3/4 located together, due to the higher butyrate production by the microbiota of donors 1/2, as opposed to higher propionate production by the microbiota of donors 3/4 (Figure S2).

**Figure 4:**
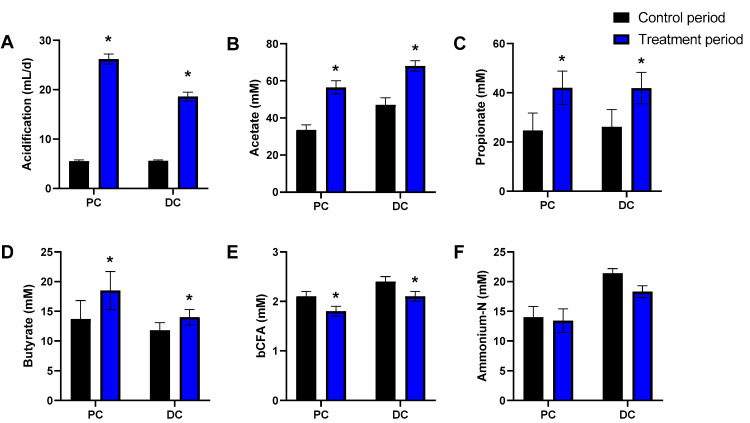
Effect of fermentation of cRG-I on microbial activity in the simulated proximal and distal colon of the quad-M-SHIME during both the control (Days 9–14) and treatment period (Days 30–35). Average (±SEM) acidification (base-acid consumption in mL/d; (**A**), acetate (**B**), propionate (**C**), butyrate (**D**), bCFA (**E**), and ammonium-N levels (**F**) across four human adult donors tested (*n* = 4). Statistically significant differences between samples collected during the final control and treatment week are indicated by * (*p* < 0.05). bCFA = branched-chain fatty acids, cRG-I = carrot-derived Rhamnogalacturonan I, DC = distal colon, PC = proximal colon.

A second key factor driving differences in the dataset was cRG-I treatment. While in both PCAs, samples of the two control weeks (C1/C2) positioned together, cRG-I treatment induced a marked shift along the PC2 axis (Figure 2A) and along both PC1/PC2 axes for the simulated PC and DC, respectively (Figure 2B). This was confirmed by the marked increase of normalized SCFA production from the first day of cRG-I treatment onwards (Day 14), for each of the four donors tested (Figure 3). The treatment effect of cRG-I became more pronounced during the first treatment week, after which it remained relatively stable. When averaging the values obtained during the final control and final treatment week (during which a steady-state was reached as visualized in Figure 3) across each of the four donors tested, cRG-I significantly increased acidification in both simulated colon regions (Figure 4A) due to the marked increase of both acetate (Figure 4), propionate (Figure 4C), and butyrate production (Figure 4D). Further, cRG-I significantly decreased bCFA levels in both PC and DC (Figure 4E).

### 3.2. cRG-I Consistently Modulated Luminal and Mucosal Microbiota Composition in the Simulated Proximal and Distal Colon of Four Human Adults as Tested in the Quad-M-SHIME

**Figure 5:**
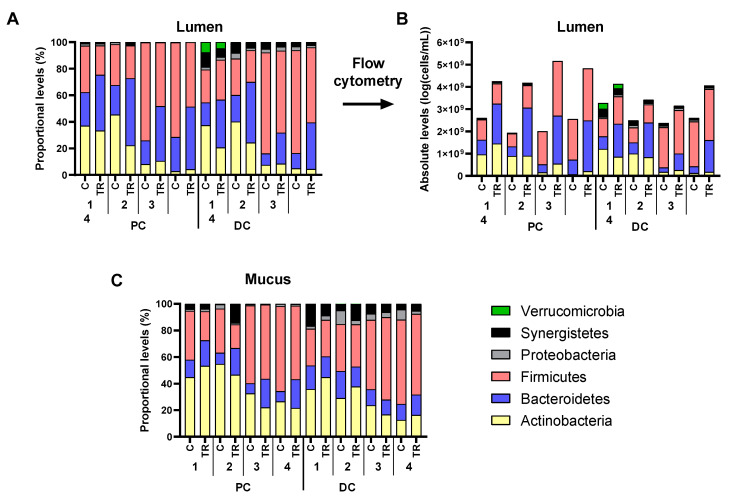
Effect of cRG-I on microbial composition (phylum level) in the lumen (log cells/mL as estimated via multiplying total cell counts (cells/mL) with 16S rRNA gene profiling (%)) (**A**,**B**) and mucus (%) (**C**) of the simulated proximal and distal colon of the quad-M-SHIME at the end of the control (C: Day 14) and treatment period (TR: Day 35). Average absolute (cells/mL as estimated via multiplying total cell counts (cells/mL) with 16S rRNA gene profiling (%)) (**A**) and proportional (%) (**B**,**C**) levels of different phyla are shown for each of the four human adult donors tested (*n* = 3 for donor 1, 2, 3, and 4; samples collected during the last control and treatment week). cRG-I = carrot-derived Rhamnogalacturonan I, DC = distal colon, PC = proximal colon.

To gain insight into microbial composition changes upon cRG-I treatment, luminal microbiota composition was first presented at phylum level, both as proportional (Figure 5A) and absolute levels (Figure 5B). This revealed that cRG-I largely increased the total number of microbial cells, in both PC and DC of all donors tested, suggesting that proportional numbers would obscure the true impact of cRG-I on microbial composition. Indeed, while *Actinobacteria,* e.g., increased in absolute numbers in the PC of Donor 1, the proportion of *Actinobacteria* in this environment decreased. Therefore, analysis of the luminal microbiota at lower taxonomic levels (family and OTU) was performed based on absolute data. In contrast, mucosal microbiota analysis was performed based on proportional data (Figure 5C) as flow cytometry analysis (as described in this paper) does not allow for accurate quantification of microbial cells in mucin-adhered microbiota [27]. 

**Figure 6:**
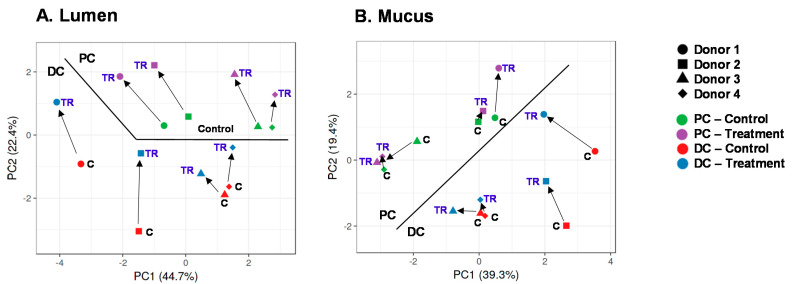
Principal component analysis (PCA) based on luminal (**A**) or mucosal (**B**) microbial composition in the proximal and distal colon of the quad-M-SHIME at the end of the control (C: Day 14) and treatment period (TR: Day 35). The PCA is based on data of the 10 most abundant families as detected via 16S-targeted Illumina sequencing across all samples. Arrows indicate the chronological evolution of samples along the experiment. cRG-I = carrot-derived Rhamnogalacturonan I, DC = distal colon, PC = proximal colon.

Like for metabolites, PCAs based on microbiota composition explained a large part of the variation in the dataset, i.e., 67.1% and 58.7% for the luminal and mucosal microbiota, respectively (Figure 6A,B). A key factor, both in the lumen and mucus, was the difference between PC and DC microbiota. Further, interindividual differences among the four donors followed from a consistent positioning of samples of a given donor along PC1, i.e., samples of donors, 1, 2, 3, and 4 clustered from left to right along PC1. The samples of donors 1/2 located together, due to the higher levels of *Bifidobacteriaceae* (~40%) and *Lachnospiraceae* (~25%), while samples of donors 3/4 clustered together given the high proportion of *Veilonellaceae* (~60%) (Table S1).

A key factor driving differences in microbial composition was cRG-I treatment. Upon initiation of cRG-I treatment, a marked shift along PC2 was observed for the luminal samples, in both PC and DC, indicating a consistent treatment effect of cRG-I on the luminal microbiota in the PC and DC regions (Figure 6A). The treatment effect of cRG-I on the luminal microbiota was confirmed to be consistent among the four donors tested, both at family (Table 1) and at OTU levels (Table 2), as illustrated by several statistically significant increases across donors (despite the noise caused by interpersonal differences in microbiome composition). A first statistically supported finding was that while overall *Bifidobacteriaceae* levels were not impacted, cRG-I strongly increased a specific OTU10 related to *B. longum*, seemingly at the expense of OTU8 related to *B. bifidum.* Further, there was a significant increase of *Bacteroidetes* upon cRG-I treatment, which was mainly driven by the marked increase of *Bacteroidaceae* and *Prevotellaceae* both in the PC and DC. At the OTU level, this could be explained by the significant increase of an OTU2 related to *Prevotella* sp. and an OTU6 related to *Bacteroides dorei*. In contrast, the levels of an OTU17 related to *Bacteroides massiliensis* decreased in the DC. Within the *Firmicutes* phylum, cRG-I significantly increased proximal levels of *Acidaminococcaceae* (~OTU11 related to *Phascolarctobacterium faecium*), *Lachnospiraceae* (~OTU9 related to *Lachnoclostridium* sp.), and *Ruminococcaceae*, while also increasing distal levels of *Erysipelotrichaceae* and *Lachnospiraceae (*~OTU5 related to *Clostridium clostridioforme*/*bolteae).* Finally, in the DC, cRG-I significantly increased *Xanthomonadaceae*, while decreasing *Ruminococcaceae* and *Synergistaceae.*

In contrast to the lumen, treatment effects on the mucosal microbiota were less pronounced (Figure 6B). Nevertheless, significant changes in the mucosal microbiota were in line with those observed in the lumen. At the OTU level, cRG-I increased OTU10 related to *B. longum* and OTU2 related to *Prevotella* sp. both in the PC and DC. This was at the expense of OTU8 (related to *B. bifidum*) in both simulated colon regions and at the expense of OTU5 (related to *Clostridium clostridioforme*/*bolteae*) in the PC. 

### 3.3. Bifidobacterium Strains Are Unable to Ferment cRG-I as Such

To assess the potential contribution of *Bifidobacterium* sp. to cRG-I fermentation, a growth experiment on solid medium was performed with single strains (Table S2). Upon 72 h incubation with glucose, all 20 strains (belonging to 10 species) were able to grow, while no growth was observed in absence of glucose. When cRG-I was provided as a sole carbon source, none of the strains showed clear growth (three strains revealed unclear growth). This supports that *Bifidobacterium* strains are unable to ferment cRG-I as such.

## 4. Discussion

Overall, the proper operation of the SHIME^®^ model during the current study followed from the stability of metabolic markers throughout the two-week control period that preceded the treatment period (Figure 2). In absence of a parallel control, such stability was required to ascribe changes during the treatment period by cRG-I treatment. On the final day of the control period, there were marked interindividual differences among donors, both at metabolic (Figure 2) and community composition level (Figure 6). The microbiota of donors 1/2 was enriched with *Bifidobacteriaceae*/*Lachnospiraceae* (Table S1) and produced higher amounts of butyrate (Figure S2). While *Bifidobacterium* sp. do not produce butyrate as such, they stimulate butyrate production by, e.g., *Lachnospiraceae* members via cross-feeding mechanisms [53,54]. In contrast, the microbiota of donors 3/4 was enriched with *Veillonellaceae* (Table S1) and produced high amounts of propionate. *Veillonella* species are indeed known to convert lactate to acetate and propionate as main end-metabolites [55]. The four donors under investigation thus allowed addressing the hypothesis whether differences in initial microbiota composition affected the outcomes of cRG-I treatment. Remarkably, cRG-I consistently stimulated microbial activity across the four donors tested with increases in average levels of acetate (+21.1 mM), propionate (+17.6 mM), and to a lesser extent also butyrate (+4.1 mM) despite baseline interindividual variability. This finding is consistent with a recent in vitro batch fermentation study that demonstrated acetate and propionate as main end-metabolites upon fermentation of cRG-I [27]. We deliberately averaged the individual microbiota data to assess if we could identify overarching changes in the microbiota composition independently of the baseline starting point and found that this was indeed the case. The consistent modulation of microbial activity and composition thus suggests that cRG-I is fermented by a specific microbial consortium. 

First, cRG-I consistently and strongly stimulated OTUs related to *B. dorei* and *Prevotella* sp. This specific increase of OTUs related to *B. dorei* and *Prevotella* sp. was also observed previously with the same compound [27]. Interestingly, when dosing pectin to in vitro fermenters, Chung et al. also identified a specific increase of *B. dorei* (detected as an OTU combined with *B. vulgatus*) amongst other members of a mixed microbiota [35]. The genera *Bacteroides* and *Prevotella* are indeed known as primary pectin degraders, possessing carbohydrate-active enzymes (CAZymes) within their polysaccharide utilization loci (PUL) [12,56]. Specifically for RG-I fermentation, *Bacteroides thetaiotaomicron* has been demonstrated to ferment RG-I via several PULs, releasing polysaccharide breakdown products in the culture medium, making them available for other microbes [25]. While several *Bacteroides* species might be capable of fermenting cRG-I, *B. dorei* seems the most capable species in the presence of a competitive background microbiota. This is supported by the genetic potential of *B. dorei* containing over 50 predicted enzymes related to pectin degradation [57]. At metabolic level, as *Bacteroides* sp. are major producers of acetate and propionate [58], the marked increase of both metabolites upon cRG-I treatment further corroborated the involvement of *Bacteroides* sp. Besides the well-described health benefits of both SCFA as reviewed by Rivière et al. [7], the presence of *B. dorei* as such could contribute to other health benefits. Inhibition of atherosclerotic lesion formation, lower endotoxemia, and suppression of proinflammatory responses have been observed in atherosclerosis-prone mice upon co-administration of live *B. vulgatus* and *B. dorei* to mice [59].

Upon cRG-I fermentation by primary degraders, polysaccharide breakdown products likely become available for other microbial species. Microbial members benefiting from such polysaccharide breakdown products included *Bifidobacterium longum* of which a related OTU strongly increased upon cRG-I treatment (+1.32 log_10_(cells/mL)). Even though the overall *Bifidobacteriaceae* family did not increase in this experiment, there was a clear stimulation of *B. longum* as previously observed in batch fermentations with cRG-I [27]. Additional growth experiments indicated that monocultures of *Bifidobacterium* species are unable to grow on cRG-I as a sole carbon source but are part of the bacterial consortium needed to ferment the complex cRG-I structure. This is in line with Chung et al., who did not identify any predicted enzymes concerned with pectin degradation in *Bifidobacteriaceae* sp. [57]. Moreover, this is also in agreement with the conclusion of Kelly et al. that the complexity of pectin is likely too high for *Bifidobacteriaceae*, rendering them dependent on the initial degradation of these large polymers by *Bacteroides* sp. [15]. The released oligo- and monosaccharides could further be then scavenged by *Bifidobacteriaceae* that indeed are capable of fermenting arabinans and galactans [60,61], both side chains of cRG-I [26]. While likely not being the primary degraders, the consistent increase of *Bifidobacteriaceae* species in this or preceding studies [27], along with the well-documented health effects of members of this family [15], support the health-promoting potential of cRG-I fermentation by the human gut microbiota.

Another microbial member that is likely part of the cRG-I fermenting consortium is *Phascolarctobacterium faecium* as an OTU related to this species increased consistently over the four donors tested (+0.47 log_10_(cells/mL)). The stimulation of this species likely followed from the succinate production of aforementioned *Prevotella* sp. [62] or *Bacteroides* sp. [58], thus boosting *Phascolarctobacterium faecium*, an abundant colonizer [63] that is able to convert succinate into propionate [64]. Besides further contributing to health effects ascribed to propionate such as exerting anti-inflammatory effects, promoting satiety, lowering blood cholesterol, decreasing liver lipogenesis, and improving insulin sensitivity (as reviewed by Rivière et al. [7]), the presence of *Phascolarctobacterium faecium* has for instance also been linked to a positive mood [65], further supporting the health-promoting potential of cRG-I fermentation by the human gut microbiota.

Other microbial families that consistently increased across the four donors tested included *Erysipelotrichaceae* (DC), *Lachnospiraceae* (PC and DC), and *Ruminococcaceae* (PC). These changes correlated with the modest increases in butyrate levels that were observed upon cRG-I treatment, butyrate being indeed produced by members belonging to these families [58]. Overall, the consistency of the changes across these families further supports the contribution of a specialized consortium in cRG-I fermentation.

As mentioned above, the first cRG-I fecal fermentation experiment was conducted in a 2-day batch incubation model [27]. Interestingly, results obtained in the batch and quad-M-SHIME in vitro models with cRG-I were consistent at metabolic and compositional levels, resulting in high levels of acetate and propionate via the involvement of *Bacteroides dorei*, *Prevotella* sp., and *Bifidobacterium longum*-related OTUs. Both studies also indicated the involvement of succinate scavengers, i.e., *Phasolarctobacterium faecium* (quad-M-SHIME) and *Dialister succinatiphilus* (batch incubation). A difference between both models was that the batch incubation strategy allowed the observation of effects of cRG-I treatment on a broader range of microbes. It was shown that cRG-I also stimulated other *Bacteroides* sp. beyond *B. dorei*, i.e., *B. ovatus*, *B. plebeius,* and *B. xylanisolvens*, while also boosting two *F. prausnitzii* OTUs and an OTU related to *R. hominis* that profoundly increased in mucus from 9.8% in the blank up to 64.3% upon cRG-I treatment (27). That the batch incubation strategy allowed to observe effects on a broader range of microbes is likely explained by the fact that in batch fermentations, in vivo-derived fecal microbiota in its maximal diversity is used as direct inoculum, an approach of which the biorelevance is supported by the minor spatial differences along the colon according to recent in vivo studies [66]. In contrast, long-term in vitro experiments involve a pre-growth of the in vivo-derived inoculum under fixed laboratory conditions for several days up to weeks (as in the current study). While this results in a stable microbiota at the start of the treatment, such pre-growth decreases the diversity by around 50%, mostly due to washout of *Clostridium* cluster IV and XIVa members (including *F. prausnitzii* and *R. hominis*) [67]. This likely explains why the treatment effects of cRG-I on *F. prausnitzii* and *R. hominis* observed in batch fermentations were not recapitulated in the quad-M-SHIME. While *Clostridium* cluster IV and XIVa members can be more optimally maintained in in vitro gut models by including a simulation of the mucosal environment, as was shown during 3-day experiments [38], frequent replacement of the mucosal beads results in loss of species-specific colonization of the mucus layer [39], likely due to limited cross-spreading between old and new mucin beads or due to physical opening of reactors to replace mucin beads. This was confirmed in the current study where despite inclusion of a mucosal microbiota simulation, no OTUs related to, e.g., *R. hominis* were detected in any of the donors at the start of the treatment (after 4 weeks of pre-growth), impairing the observation of treatment effects on such mucosal microbes. The washout of true mucosal microbes upon long pre-growth periods could potentially also explain why treatment effects on lumen and mucus were relatively similar during the current study. Despite such differences between both in vitro models, many findings were consistent and provided confidence in the robustness of (i) the observed impact of cRG-I on the human gut microbiota and (ii) the use of both types of in vitro gut models in gut microbiome research. While the added value of in vitro gastro-intestinal models is currently well-recognized [68], it is acknowledged that they mimic only partly the complexity of the gut ecosystem. Nevertheless, confidence in both in vitro gut models is further provided by the observation of similar shifts in microbial community composition during a recent clinical trial in which RG-I was dosed at 0.3 or 1.5 g/day to the daily diet (manuscript in preparation).

Finally, a critical remark on the limitation of the applied experimental design is that the reference to which treatment effects were compared consisted of a preceding control period. Such longitudinal control is common in a SHIME setup [69,70,71], and the stability of in vitro communities upon applying a 2-week stabilization period has previously been demonstrated [67]. It would have been ideal to follow the stability of the microbial communities during the entire duration of the current study by means of incorporating four additional untreated parallel control arms. As the purpose of the present study was to assess the consistency of the impact of cRG-I on individual fecal samples, such a control would not optimally be performed with a single pooled fecal sample. It was therefore important to start with well-characterized and stable individual microbial communities.

## 5. Conclusions

Overall, this study confirms the potential of cRG-I to beneficially modulate the human gut microbiome with a marked consistency in SCFA production and microbial modulation across simulated human subjects displaying individual variability at baseline. High amounts of acetate and propionate were produced due to the involvement of a specialized consortium consisting of, amongst others, strains belonging to *Bacteroides dorei*, *Prevotella* sp., and *Bifidobacterium longum.* Due to its structural complexity, cRG-I corresponds to a unique polysaccharide demonstrating prebiotic properties supported by a concomitant effect on the host microbiota and immune system (26). As a next step, it will be necessary to link the changes in microbiota composition and metabolite production to a discrete health benefit in well-designed human trials and altogether verify the predictivity of the data generated in vitro in the described study.

## Figures and Tables

**Table 1:** Effect of cRG-I on microbial composition at family level in the lumen (log cells/mL as estimated via multiplying total cell counts (cells/mL) with 16S rRNA gene profiling (%)) and mucus (%) of the simulated proximal (PC) and distal colon (DC) of the quad-M-SHIME. Values presented are the averages of the differences at the end of the treatment period (Day 35) versus those at the end of the control period (Day 14) across the four human adult donors tested (*n* = 4). As a result, positive or negative values indicate families that are increased or decreased, respectively, upon cRG-I treatment. Statistical differences are indicated in bold.

Phylum	Family	Lumen		Mucus	
		PC	DC	PC	DC
Actinobacteria	*Atopobiaceae*	0.43	0.30	0.11%	0.02%
	*Bifidobacteriaceae*	0.30	0.02	−6.00%	3.98%
	*Coriobacteriaceae*	0.23	−0.02	2.16%	−0.41%
	*Eggerthellaceae*	0.00	−0.17	0.00%	−0.02%
Bacteroidetes	*Bacteroidaceae*	**0.61**	**0.33**	**3.99%**	**−2.81%**
	*Barnesiellaceae*	0.00	0.27	0.00%	0.05%
	*Marinifilaceae*	0.00	0.01	0.00%	0.01%
	*Muribaculaceae*	0.00	−0.01	0.00%	0.02%
	*Prevotellaceae*	0.52	**0.69**	**7.55%**	**1.85%**
	*Rikenellaceae*	−0.02	0.07	**−0.07%**	−0.30%
	*Tannerellaceae*	0.38	−0.10	−0.12%	−0.03%
Firmicutes	*Acidaminococcaceae*	**0.47**	0.39	0.46%	0.16%
	*Christensenellaceae*	0.00	0.44	0.01%	0.21%
	*Clostridiaceae*	0.00	0.00	−0.01%	−1.00%
	*Erysipelotrichaceae*	0.16	**0.68**	0.22%	0.18%
	*Family XIII*	0.00	0.01	0.00%	−0.03%
	*Lachnospiraceae*	**0.24**	**0.19**	−3.83%	0.96%
	*Lactobacillaceae*	0.00	0.03	−0.15%	0.02%
	*Ruminococcaceae*	**0.43**	**−0.21**	0.08%	**−1.15%**
	*Staphylococcaceae*	0.03	0.19	**0.01%**	0.07%
	*Veillonellaceae*	−0.08	−0.13	**−7.19%**	1.41%
Proteobacteria	*Burkholderiaceae*	0.18	0.12	−0.36%	−0.36%
	*Desulfovibrionaceae*	−0.28	−0.06	**−0.60%**	**−0.75%**
	*Enterobacteriaceae*	0.06	0.25	0.32%	−0.27%
	*Pseudomonadaceae*	0.00	0.00	−0.01%	0.00%
	*uncultured*	0.00	0.64	0.00%	0.20%
	*Xanthomonadaceae*	0.39	**0.42**	0.04%	0.00%
Synergistetes	*Synergistaceae*	0.54	**−0.09**	3.36%	−0.22%
Verrucomicrobia	*Akkermansiaceae*	0.00	−0.09	0.00%	−0.01%

**Table 2:** Effect of cRG-I on microbial composition (25 most abundant OTUs across all samples) in the lumen (log cells/mL as estimated via multiplying total cell counts (cells/mL) with 16S rRNA gene profiling (%)) and mucus (%) of the simulated proximal (PC) and distal colon (DC) of the quad-M-SHIME. Values presented are the averages of the differences at the end of the treatment period (Day 35) versus those at the end of the control period (Day 14) across the four human adult donors tested (*n* = 4). As a result, positive values indicate that a given OTU is increased upon cRG-I treatment. Statistical differences are indicated in bold.

Phylum	Family	OTU	Related Species	Lumen		Mucus	
				PC	DC	PC	DC
Actinobacteria	*Bifidobacteriaceae*	1	*Bifidobacterium adolescentis*	0.26	−0.01	−1.03%	6.64%
		8	*Bifidobacterium bifidum*	**−0.56**	**−0.84**	**−9.28%**	**−5.96%**
		10	*Bifidobacterium longum*	**1.32**	**1.05**	**4.13%**	**3.23%**
	*Coriobacteriaceae*	12	*Collinsella aerofaciens*	0.23	−0.02	2.16%	−0.41%
Bacteroidetes	*Bacteroidaceae*	6	*Bacteroides dorei*	**0.83**	**0.73**	5.22%	−0.14%
		16	*Bacteroides ovatus*	−0.38	0.03	−0.22%	0.04%
		28	*Bacteroides fragilis*	−0.58	−0.80	−0.29%	−2.09%
		17	*Bacteroides massiliensis*	−0.25	**−0.57**	−1.12%	−1.25%
		13	*Bacteroides intestinalis*	−0.19	0.02	−0.17%	0.20%
	*Prevotellaceae*	2	*Prevotella* sp.	0.52	**0.69**	**7.55%**	**1.85%**
Firmicutes	*Acidaminococcaceae*	11	*Phascolarctobacterium faecium*	**0.47**	0.39	0.46%	0.16%
	*Clostridiaceae*	32	*Clostridium butyricum*	0.00	0.00	−0.01%	−0.98%
	*Lachnospiraceae*	5	*Clostridium clostridioforme*/*bolteae*	0.04	**0.15**	**−3.74%**	2.27%
		9	*Lachnoclostridium* sp.	**0.57**	0.16	−2.16%	−0.09%
		14	*Eubacterium contortum*	0.00	0.04	0.00%	−1.44%
		25	*Clostridium* sp.	0.34	0.32	0.10%	0.11%
	*Ruminococcaceae*	19	*Gemmiger formicilis*	0.36	−0.05	0.06%	−0.74%
		42	*Faecalibacterium prausnitzii*	0.00	0.00	0.00%	−0.10%
	*Veillonellaceae*	3	*Megamonas funiformis*	0.05	−0.01	−1.70%	0.52%
		4	*Megamonas funiformis*	0.06	0.01	−0.26%	1.21%
		18	*Megasphaera* sp.	−0.05	−0.13	−1.78%	−0.02%
		26	*Anaeroglobus geminatus*	−0.01	−0.24	−1.49%	−0.21%
Proteobacteria	*Pseudomonadaceae*	15	*Pseudomonas aeruginosa*	0.31	−0.06	0.04%	−1.88%
Synergistetes	*Synergistaceae*	7	*Cloacibacillus* sp.	0.54	**−0.09**	3.36%	0.04%
Verrucomicrobia	*Akkermansiaceae*	20	*Akkermansia muciniphila*	0.00	−0.09	0.00%	−0.01%

## Supplementary Materials

The following are available online at https://www.mdpi.com/article/10.3390/microorganisms9102142/s1, Figure S1. Effect of fermentation of cRG-I on SCFA production for donors 2 and 4, Figure S2. Effect of fermentation of cRG-I on absolute SCFA levels for donors 1, 2, 3 and 4, Table S1. Microbial composition in the lumen (%) of the simulated proximal and distal colon of the quad-M-SHIME, Table S2. Growth of single *Bifidobacterium* strains in LMG medium 144 with glucose (positive control), without carbon source (negative control), and with cRG-I (0.5% *w*/*v*).

## References

[1] Marchesi J.R., Adams D.H., Fava F., Hermes G.D.A., Hirschfield G.M., Hold G., Quraishi M.N., Kinross J., Smidt H., Tuohy K.M. (2016). The gut microbiota and host health: A new clinical frontier. Gut.

[2] Boulangé C.L., Neves A.L., Chilloux J., Nicholson J.K., Dumas M.-E. (2016). Impact of the gut microbiota on inflammation, obesity, and metabolic disease. Genome Med..

[3] Tilg H., Cani P.D., Mayer E.A. (2016). Gut microbiome and liver diseases. Gut.

[4] Ni J., Wu G.D., Albenberg L., Tomov V.T. (2017). Gut microbiota and IBD: Causation or correlation?. Nat. Rev. Gastroenterol. Hepatol..

[5] Ternes D., Karta J., Tsenkova M., Wilmes P., Haan S., Letellier E. (2020). Microbiome in Colorectal Cancer: How to Get from Meta-omics to Mechanism?. Trends Microbiol..

[6] Flint H.J., Scott K.P., Louis P., Duncan S.H. (2012). The role of the gut microbiota in nutrition and health. Nat. Rev. Gastroenterol. Hepatol..

[7] Rivière A., Selak M., Lantin D., Leroy F., De Vuyst L. (2016). Bifidobacteria and Butyrate-Producing Colon Bacteria: Importance and Strategies for Their Stimulation in the Human Gut. Front. Microbiol..

[8] Koh A., De Vadder F., Kovatcheva-Datchary P., Bäckhed F. (2016). From Dietary Fiber to Host Physiology: Short-Chain Fatty Acids as Key Bacterial Metabolites. Cell.

[9] Gonçalves P., Araújo J.R., Di Santo J.P. (2018). A Cross-Talk Between Microbiota-Derived Short-Chain Fatty Acids and the Host Mucosal Immune System Regulates Intestinal Homeostasis and Inflammatory Bowel Disease. Inflamm. Bowel Dis..

[10] van der Hee B., Wells J.M. (2021). Microbial Regulation of Host Physiology by Short-chain Fatty Acids. Trends Microbiol..

[11] Ndeh D., Gilbert H.J. (2018). Biochemistry of complex glycan depolymerisation by the human gut microbiota. FEMS Microbiol. Rev..

[12] Martens E.C., Lowe E.C., Chiang H., Pudlo N.A., Wu M., McNulty N.P., Abbott D.W., Henrissat B., Gilbert H.J., Bolam D.N. (2011). Recognition and Degradation of Plant Cell Wall Polysaccharides by Two Human Gut Symbionts. PLoS Biol..

[13] Cuskin F., Lowe E.C., Temple M.J., Zhu Y., Cameron E., Pudlo N.A., Porter N.T., Urs K., Thompson A.J., Cartmell A. (2015). Human gut Bacteroidetes can utilize yeast mannan through a selfish mechanism. Nature.

[14] Larsbrink J., Rogers T.E., Hemsworth G.R., McKee L.S., Tauzin A.S., Spadiut O., Klinter S., Pudlo N.A., Urs K., Koropatkin N.M. (2014). A discrete genetic locus confers xyloglucan metabolism in select human gut Bacteroidetes. Nature.

[15] Kelly S.M., Munoz-Munoz J., van Sinderen D. (2021). Plant Glycan Metabolism by Bifidobacteria. Front. Microbiol..

[16] Windey K., De Preter V., Verbeke K. (2012). Relevance of protein fermentation to gut health. Mol. Nutr. Food Res..

[17] Gibson G.R., Hutkins R., Sanders M.E., Prescott S.L., Reimer R.A., Salminen S.J., Scott K., Stanton C., Swanson K.S., Cani P.D. (2017). Expert consensus document: The International Scientific Association for Probiotics and Prebiotics (ISAPP) consensus statement on the definition and scope of prebiotics. Nat. Rev. Gastroenterol. Hepatol..

[18] Sabater-Molina M., Larqué E., Torrella F., Zamora S. (2009). Dietary fructooligosaccharides and potential benefits on health. J. Physiol. Biochem..

[19] Le Bastard Q., Chapelet G., Javaudin F., Lepelletier D., Batard E., Montassier E. (2020). The effects of inulin on gut microbial composition: A systematic review of evidence from human studies. Eur. J. Clin. Microbiol. Infect. Dis..

[20] Bode L. (2009). Human milk oligosaccharides: Prebiotics and beyond. Nutr. Rev..

[21] Broekaert W.F., Courtin C.M., Verbeke K., Van de Wiele T., Verstraete W., Delcour J.A. (2011). Prebiotic and other health-related effects of cereal-derived arabinoxylans, arabinoxylan-oligosaccharides, and xylooligosaccharides. Crit. Rev. Food Sci. Nutr..

[22] Tingirikari J.M.R. (2019). In-Vitro Prebiotic Analysis of Microbiota Accessible Pectic Polysaccharides. Curr. Microbiol..

[23] Wu D., Zheng J., Mao G., Hu W., Ye X., Linhardt R.J., Chen S. (2020). Rethinking the impact of RG-I mainly from fruits and vegetables on dietary health. Crit. Rev. Food Sci. Nutr..

[24] Wu D., Ye X., Linhardt R.J., Liu X., Zhu K., Yu C., Ding T., Liu D., He Q., Chen S. (2021). Dietary pectic substances enhance gut health by its polycomponent: A review. Compr. Rev. Food Sci. Food Saf..

[25] Luis A.S., Briggs J., Zhang X., Farnell B., Ndeh D., Labourel A., Baslé A., Cartmell A., Terrapon N., Stott K. (2018). Dietary pectic glycans are degraded by coordinated enzyme pathways in human colonic Bacteroides. Nat. Microbiol..

[26] McKay S., Oranje P., Helin J., Koek J.H., Kreijveld E., van den Abbeele P., Pohl U., Bothe G., Tzoumaki M., Aparicio-Vergara M. (2021). Development of an Affordable, Sustainable and Efficacious Plant-Based Immunomodulatory Food Ingredient Based on Bell Pepper or Carrot RG-I Pectic Polysaccharides. Nutrients.

[27] Van den Abbeele P., Verstrepen L., Ghyselinck J., Albers R., Marzorati M., Mercenier A. (2020). A Novel Non-Digestible, Carrot-Derived Polysaccharide (cRG-I) Selectively Modulates the Human Gut Microbiota while Promoting Gut Barrier Integrity: An Integrated In Vitro Approach. Nutrients.

[28] Ruppin H., Bar-Meir S., Soergel K.H., Wood C.M., Schmitt M.G. (1980). Absorption of Short-Chain Fatty Acids by the Colon. Gastroenterology.

[29] Boets E., Deroover L., Houben E., Vermeulen K., Gomand S.V., Delcour J.A., Verbeke K. (2015). Quantification of in Vivo Colonic Short Chain Fatty Acid Production from Inulin. Nutrients.

[30] Sakata T. (2019). Pitfalls in short-chain fatty acid research: A methodological review. Anim. Sci. J..

[31] Chen T., Long W., Zhang C., Liu S., Zhao L., Hamaker B.R. (2017). Fiber-utilizing capacity varies in Prevotella—versus Bacteroides—dominated gut microbiota. Sci. Rep..

[32] Molly K., Woestyne M.V., Smet I.D., Verstraete W. (1994). Validation of the Simulator of the Human Intestinal Microbial Ecosystem (SHIME) Reactor Using Microorganism-associated Activities. Microb. Ecol. Health Dis..

[33] Minekus M., Smeets-Peeters M., Bernalier A., Marol-Bonnin S., Havenaar R., Marteau P., Alric M., Fonty G., Huis in’t Veld J.H.J. (1999). A computer-controlled system to simulate conditions of the large intestine with peristaltic mixing, water absorption and absorption of fermentation products. Appl. Microbiol. Biotechnol..

[34] Berner A.Z., Fuentes S., Dostal A., Payne A.N., Gutierrez P.V., Chassard C., Grattepanche F., de Vos W.M., Lacroix C. (2013). Novel Polyfermentor Intestinal Model (PolyFermS) for Controlled Ecological Studies: Validation and Effect of pH. PLoS ONE.

[35] Chung W.S.F., Walker A.W., Louis P., Parkhill J., Vermeiren J., Bosscher D., Duncan S.H., Flint H.J. (2016). Modulation of the human gut microbiota by dietary fibres occurs at the species level. BMC Biol..

[36] Salonen A., Lahti L., Salojärvi J., Holtrop G., Korpela K., Duncan S.H., Date P., Farquharson F., Johnstone A.M., Lobley G.E. (2014). Impact of diet and individual variation on intestinal microbiota composition and fermentation products in obese men. ISME J..

[37] Healey G.R., Murphy R., Brough L., Butts C.A., Coad J. (2017). Interindividual variability in gut microbiota and host response to dietary interventions. Nutr. Rev..

[38] Van den Abbeele P., Belzer C., Goossens M., Kleerebezem M., De Vos W.M., Thas O., De Weirdt R., Kerckhof F.-M., Van de Wiele T. (2013). Butyrate-producing Clostridium cluster XIVa species specifically colonize mucins in an in vitro gut model. ISME J..

[39] Van den Abbeele P., Marzorati M., Derde M., De Weirdt R., Joan V., Possemiers S., Van de Wiele T. (2016). Arabinoxylans, inulin and Lactobacillus reuteri 1063 repress the adherent-invasive Escherichia coli from mucus in a mucosa-comprising gut model. NPJ Biofilms Microbiomes.

[40] Moens F., Duysburgh C., van den Abbeele P., Morera M., Marzorati M. (2019). Lactobacillus rhamnosus GG and Saccharomyces cerevisiae boulardii exert synergistic antipathogenic activity in vitro against enterotoxigenic Escherichia coli. Benef. Microbes.

[41] De Weirdt R., Possemiers S., Vermeulen G., Moerdijk-Poortvliet T.C.W., Boschker H.T.S., Verstraete W., Van de Wiele T. (2010). Human faecal microbiota display variable patterns of glycerol metabolism. FEMS Microbiol. Ecol..

[42] Van de Wiele T., Boon N., Possemiers S., Jacobs H., Verstraete W. (2004). Prebiotic effects of chicory inulin in the simulator of the human intestinal microbial ecosystem. FEMS Microbiol. Ecol..

[43] Boon N., Top E.M., Verstraete W., Siciliano S.D. (2003). Bioaugmentation as a Tool To Protect the Structure and Function of an Activated-Sludge Microbial Community against a 3-Chloroaniline Shock Load. Appl. Environ. Microbiol..

[44] Duysburgh C., Van den Abbeele P., Krishnan K., Bayne T.F., Marzorati M. (2019). A synbiotic concept containing spore-forming Bacillus strains and a prebiotic fiber blend consistently enhanced metabolic activity by modulation of the gut microbiome in vitro. Int. J. Pharm. X.

[45] Schloss P.D., Westcott S.L. (2011). Assessing and Improving Methods Used in Operational Taxonomic Unit-Based Approaches for 16S rRNA Gene Sequence Analysis. Appl. Environ. Microbiol..

[46] Kozich J.J., Westcott S.L., Baxter N.T., Highlander S.K., Schloss P.D. (2013). Development of a Dual-Index Sequencing Strategy and Curation Pipeline for Analyzing Amplicon Sequence Data on the MiSeq Illumina Sequencing Platform. Appl. Environ. Microbiol..

[47] Wang Q., Garrity G.M., Tiedje J.M., Cole J.R. (2007). Naive Bayesian classifier for rapid assignment of rRNA sequences into the new bacterial taxonomy. Appl. Environ. Microbiol..

[48] Cole J.R., Wang Q., Cardenas E., Fish J., Chai B., Farris R.J., Kulam-Syed-Mohideen A.S., McGarrell D.M., Marsh T., Garrity G.M. (2009). The Ribosomal Database Project: Improved alignments and new tools for rRNA analysis. Nucleic Acids Res..

[49] Hammer O., Harper D.A.T., Ryan P.D. (2001). PAST: Paleontological Statistics Software Package for Education and Data Analysis. Palaeontol. Electron..

[50] Vandeputte D., Kathagen G., D’hoe K., Vieira-Silva S., Valles-Colomer M., Sabino J., Wang J., Tito R.Y., De Commer L., Darzi Y. (2017). Quantitative microbiome profiling links gut community variation to microbial load. Nature.

[51] Metsalu T., Vilo J. (2015). ClustVis: A web tool for visualizing clustering of multivariate data using Principal Component Analysis and heatmap. Nucleic Acids Res..

[52] Lee S., Lee D.K. (2018). What is the proper way to apply the multiple comparison test?. Korean J. Anesthesiol..

[53] Falony G., Vlachou A., Verbrugghe K., Vuyst L.D. (2006). Cross-Feeding between Bifidobacterium longum BB536 and Acetate-Converting, Butyrate-Producing Colon Bacteria during Growth on Oligofructose. Appl. Environ. Microbiol..

[54] Rios-Covian D., Gueimonde M., Duncan S.H., Flint H.J., de los Reyes-Gavilan C.G. (2015). Enhanced butyrate formation by cross-feeding between Faecalibacterium prausnitzii and Bifidobacterium adolescentis. FEMS Microbiol. Lett..

[55] Ng S.K.C., Hamilton I.R. (1971). Lactate Metabolism by Veillonella parvula. J. Bacteriol..

[56] Ndeh D., Rogowski A., Cartmell A., Luis A.S., Baslé A., Gray J., Venditto I., Briggs J., Zhang X., Labourel A. (2017). Complex pectin metabolism by gut bacteria reveals novel catalytic functions. Nature.

[57] Chung W.S.F., Meijerink M., Zeuner B., Holck J., Louis P., Meyer A.S., Wells J.M., Flint H.J., Duncan S.H. (2017). Prebiotic potential of pectin and pectic oligosaccharides to promote anti-inflammatory commensal bacteria in the human colon. FEMS Microbiol. Ecol..

[58] Louis P., Flint H.J. (2017). Formation of propionate and butyrate by the human colonic microbiota. Environ. Microbiol..

[59] Yoshida N., Emoto T., Yamashita T., Watanabe H., Hayashi T., Tabata T., Hoshi N., Hatano N., Ozawa G., Sasaki N. (2018). Bacteroides vulgatus and Bacteroides dorei Reduce Gut Microbial Lipopolysaccharide Production and Inhibit Atherosclerosis. Circulation.

[60] O’Connell Motherway M., Fitzgerald G.F., van Sinderen D. (2011). Metabolism of a plant derived galactose-containing polysaccharide by Bifidobacterium breve UCC2003. Microb. Biotechnol..

[61] Komeno M., Hayamizu H., Fujita K., Ashida H. (2019). Two Novel α-l-Arabinofuranosidases from Bifidobacterium longum subsp. longum Belonging to Glycoside Hydrolase Family 43 Cooperatively Degrade Arabinan. Appl. Environ. Microbiol..

[62] Franke T., Deppenmeier U. (2018). Physiology and central carbon metabolism of the gut bacterium Prevotella copri. Mol. Microbiol..

[63] Wu F., Guo X., Zhang J., Zhang M., Ou Z., Peng Y. (2017). Phascolarctobacterium faecium abundant colonization in human gastrointestinal tract. Exp. Ther. Med..

[64] Dot T., Osawa R., Stackebrandt E. (1993). Phascolarctobacterium faecium gen. nov, spec. nov., a Novel Taxon of the Sporomusa Group of Bacteria. Syst. Appl. Microbiol..

[65] Li L., Su Q., Xie B., Duan L., Zhao W., Hu D., Wu R., Liu H. (2016). Gut microbes in correlation with mood: Case study in a closed experimental human life support system. Neurogastroenterol. Motil. Off. J. Eur. Gastrointest. Motil. Soc..

[66] Lavelle A., Lennon G., O’Sullivan O., Docherty N., Balfe A., Maguire A., Mulcahy H.E., Doherty G., O’Donoghue D., Hyland J. (2015). Spatial variation of the colonic microbiota in patients with ulcerative colitis and control volunteers. Gut.

[67] Van den Abbeele P., Grootaert C., Marzorati M., Possemiers S., Verstraete W., Gérard P., Rabot S., Bruneau A., El Aidy S., Derrien M. (2010). Microbial community development in a dynamic gut model is reproducible, colon region specific, and selective for Bacteroidetes and Clostridium cluster IX. Appl. Environ. Microbiol..

[68] Dupont D., Alric M., Blanquet-Diot S., Bornhorst G., Cueva C., Deglaire A., Denis S., Ferrua M., Havenaar R., Lelieveld J. (2019). Can dynamic in vitro digestion systems mimic the physiological reality?. Crit. Rev. Food Sci. Nutr..

[69] Šuligoj T., Vigsnæs L.K., den Abbeele P.V., Apostolou A., Karalis K., Savva G.M., McConnell B., Juge N. (2020). Effects of Human Milk Oligosaccharides on the Adult Gut Microbiota and Barrier Function. Nutrients.

[70] Marzorati M., Van den Abbeele P., Bubeck S., Bayne T., Krishnan K., Young A. (2021). Treatment with a spore-based probiotic containing five strains of Bacillus induced changes in the metabolic activity and community composition of the gut microbiota in a SHIME^®^ model of the human gastrointestinal system. Food Res. Int..

[71] Moens F., Van den Abbeele P., Basit A.W., Dodoo C., Chatterjee R., Smith B., Gaisford S. (2019). A four-strain probiotic exerts positive immunomodulatory effects by enhancing colonic butyrate production in vitro. Int. J. Pharm..

