# Supplementary material for: Consistent Prebiotic Effects of Carrot RG-I on the Gut Microbiota of Four Human Adult Donors in the SHIME® Model despite Baseline Individual Variability
Source: Microorganisms. 2021 Oct 13;9(10):2142. doi: 10.3390/microorganisms9102142 (PMC8538933; doi:10.3390/microorganisms9102142)
Supplement: Supplementary file 1 [file microorganisms-09-02142-s001.zip › microorganisms-1389152-supplementary.pdf]

microorganisms

MDPI

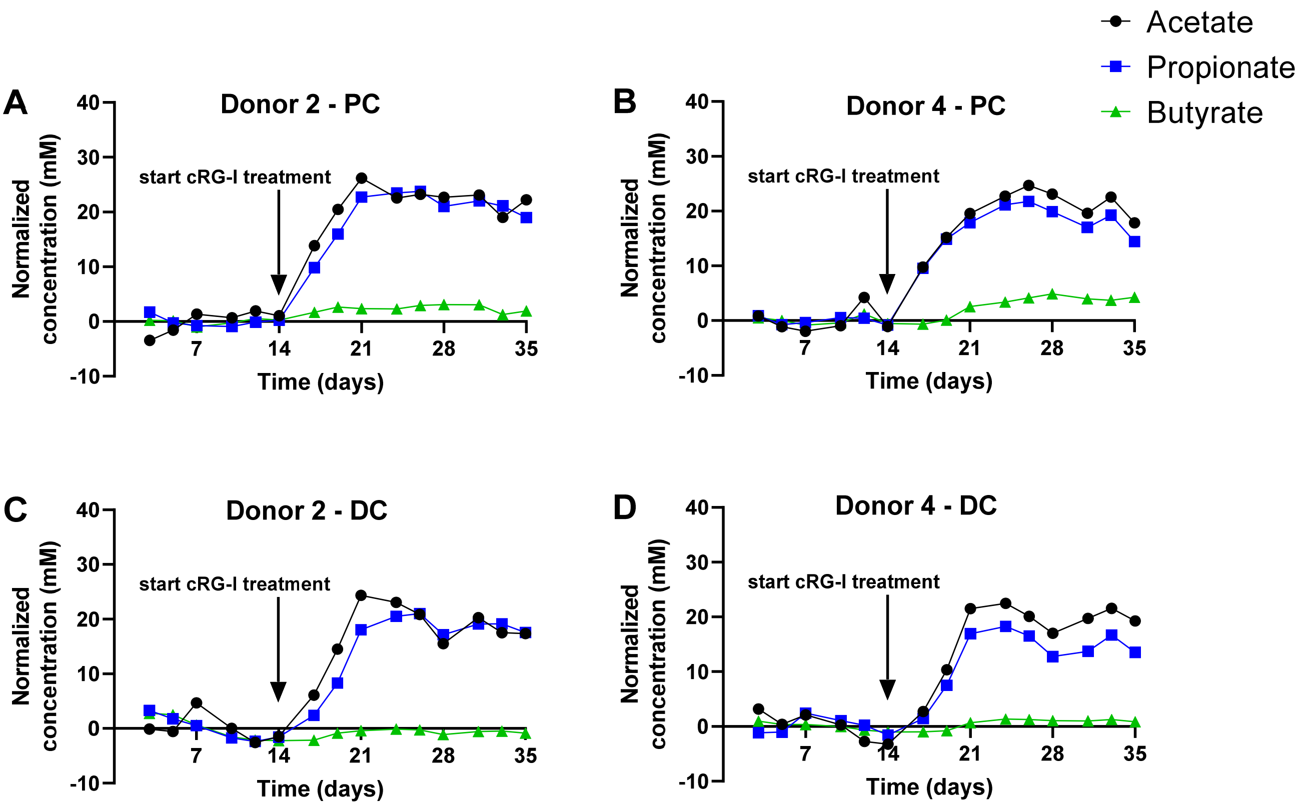

Figure S1. Effect of fermentation of cRG-I on SCFA production in the simulated proximal (pannels A/B) and distal colon (pannels C/D) during the control (days 0-14) and treatment periods (days 14-35) of the quad-M-SHIME simulating donors 2 (pannels A/C) and 4 (pannels B/D). Data are expressed as normalized values versus the average of the control period. Arrows indicate the start of the cRG-I treatment. cRG-I = carrot-derived Rhamnogalacturonan I, DC = distal colon, PC = proximal colon. The absolute values on which the normalized concentrations are based are shown in Figure S2 B/D/F/H.

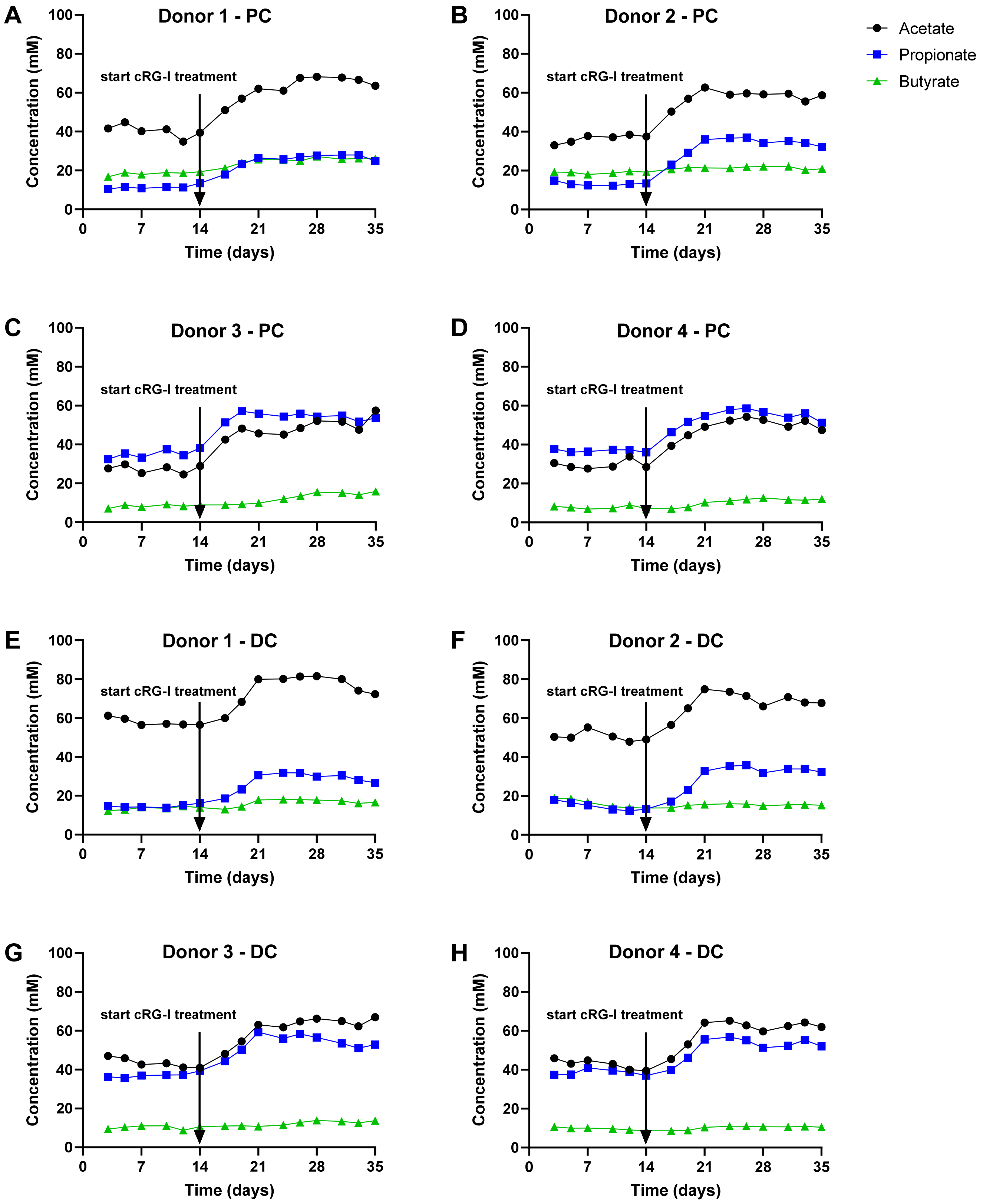

Figure S2. Effect of fermentation of cRG-I on absolute SCFA levels in the simulated proximal (pannels A/B/C/D) and distal (pannels E/F/G/H) colon during the control (days 0-14) and treatment periods (days 14-35) of the quad-M-SHIME simulating donors 1 (pannels A/E), 2 (pannels B/F), 3 (pannels C/G) and 4 (pannels D/H). Arrows indicate the start of the cRG-I treatment. cRG-I = carrot-derived Rhamnogalacturonan I, DC = distal colon, PC = proximal colon.

Table S1. Microbial composition (10 most abundant families across all samples) in the lumen (%) of the simulated proximal (PC) and distal colon (DC) of the quad-M-SHIME. Values presented are the averages at the end of the control period (day 14) for each of the four human adult donors (donors 1, 2, 3 and 4) tested in technical triplicate.

Family	PC				DC			
	1	2	3	4	1	2	3	4
Bifidobacteriaceae	36.8%	45.5%	8.0%	2.7%	36.9%	39.4%	7.5%	4.7%
Bacteroidaceae	24.9%	5.5%	2.5%	1.0%	16.3%	7.1%	3.4%	6.0%
Prevotellaceae	0.0%	16.4%	15.2%	24.7%	0.0%	11.5%	1.8%	3.4%
Acidaminococcaceae	1.1%	2.7%	0.2%	0.0%	0.9%	2.8%	0.6%	0.4%
Lachnospiraceae	24.8%	26.1%	12.3%	8.2%	21.7%	15.1%	12.3%	12.4%
Ruminococcaceae	0.1%	0.1%	0.0%	0.1%	1.6%	8.5%	6.0%	2.7%
Veillonellaceae	9.1%	1.1%	61.3%	62.8%	0.3%	0.2%	56.9%	62.0%
Desulfovibrionaceae	1.1%	0.7%	0.0%	0.0%	0.8%	1.8%	0.7%	1.2%
Synergistaceae	1.2%	0.1%	0.0%	0.0%	11.1%	8.0%	5.2%	3.2%
Akkermansiaceae	0.0%	0.0%	0.0%	0.0%	7.9%	0.3%	0.0%	0.2%

Table S2. Growth of single *Bifidobacterium* strains in LMG medium 144 containing glucose (positive control), no carbon source (negative control) or with cRG-I instead of glucose. Growth was scored with + and ++, with unclear growth being indicated by ?. T designates Type strains**.**

Test strain			Carbon source		
**Species**	**LMG number**	**Type**	**Glucose**	**-**	**cRG-I**
Bifidobacterium adolescentis	LMG 10502	T	++	-	-
	LMG 10733		++	-	-
	LMG 30678		+	-	-
Bifidobacterium angulatum	LMG 11039	T	+	-	-
Bifidobacterium animalis lactis	LMG 18314	T	++	-	-
	LMG 25753		++	-	-
Bifidobacterium bifidum	LMG 11041	T	+	-	?
	LMG 25759		++	-	?
Bifidobacterium breve	LMG 13194		+	-	-
	LMG 13208	T	+	-	-
	LMG 25761		++	-	-
Bifidobacterium catenulatum	LMG 11043	T	++	-	-
Bifidobacterium longum infantis	LMG 11570		++	-	-
	LMG 25762		++	-	-
	LMG 8811	T	++	-	-
Bifidobacterium longum longum	LMG 13196		++	-	-
	LMG 13197	T	++	-	-
	LMG 25763		++	-	-
Bifidobacterium longum suis	LMG 21814	T	++	-	-
Bifidobacterium pseudocatenulatum	LMG 10505	T	+	-	?
